# Circular RNA Microarray Analyses in Hepatic Ischemia-Reperfusion Injury With Ischemic Preconditioning Prevention

**DOI:** 10.3389/fmed.2021.626948

**Published:** 2021-03-08

**Authors:** Xinyao Tian, Yan Hu, Yuanxing Liu, Zhe Yang, Haiyang Xie, Lin Zhou, Shusen Zheng

**Affiliations:** ^1^Division of Hepatobiliary and Pancreatic Surgery, Department of Surgery, The First Affiliated Hospital, Zhejiang University School of Medicine, Hangzhou, China; ^2^Key Laboratory of Combined Multi-organ Transplantation, National Health Commission of PRC, Hangzhou, China; ^3^Department of Pharmacy, Second Affiliated Hospital of Dalian Medical University, Dalian, China; ^4^Department of Hepatobiliary and Pancreatic Surgery, Department of Liver Transplantation, Shulan (Hangzhou) Hospital, Hangzhou, China

**Keywords:** circular RNA, ischemia-reperfusion injury, ischemic preconditioning, microarray analyses, high-throughput sequencing

## Abstract

Ischemic preconditioning (IPC) represents an effective intervention to relieve hepatic ischemia-reperfusion injury (IRI). Systematic detection of circRNA expression revealing the protection effect of IPC still remains to be elucidated. Here, we applied a microarray to detect circRNA and mRNA expression in ischemic liver with and without IPC (*n* = 3 in each group). Compared with the sham group, there were 39 circRNAs and 432 mRNAs increased and 38 circRNAs and 254 mRNAs decreased (fold change ≥1.5, *P* < 0.05) in the group of hepatic IRI. As the result of IPC intervention, 43 circRNAs and 64 mRNAs were increased, and 7 circRNAs and 31 mRNAs were decreased in the IPC group when compared with IRI. We then identified circRNA_017753 as the most possible target that may closely relate to IPC protective signaling and predicted Jade1 as the target related to circRNA_017753. Three possible circRNA–miRNA–mRNA axes were constructed that may play a vital role in protective mechanisms in IPC. The study for the first time systematically detects the dysregulated circRNAs and mRNAs in response to hepatic IRI and IPC intervention. Our profile and bioinformatic analysis provide numerous novel clues to understanding the pathophysiologic mechanism of IPC protection against hepatic IRI.

## Introduction

Hepatic ischemia-reperfusion injury (IRI) occurs in clinical circumstances, including hepatic resection, transplantation, liver trauma, or septic shock (1). The reperfusion aggravates hepatic injury after ischemia. Concerning mechanisms involve microcirculatory failure, inflammatory cytokine release, and reactive oxygen species accumulation (2, 3). Especially in the surgical procedures of hepatic resections and liver transplantation, IRI not only contributes to organ damage, but also reduces the long-term survival rates. Therefore, strategies to reduce hepatic IRI and improve patient outcomes is clinically important at any point.

Ischemic preconditioning (IPC) refers to an intrinsic procedure in the form of repeated short episodes of ischemia that increase the resistance of organs against IRI. This powerful intervention for protection against IRI was first reported in 1986 by Murry et al. (4). Though, today, the surgical techniques and clinical conditions improve rapidly concerning the intervention of IPC, the precise molecular mechanisms behind IRI and the protection effect of IPC still remain an important problem. Moreover, recent studies comparing different protective methods of IRI have demonstrated variable protective mechanisms in IPC and ischemic postconditioning (IPostC) (5, 6). The mechanism of comparative and collaborative research on IPC and IPostC are of great significance in the research on IRI.

Nowadays, high-throughput RNA sequencing and microarray of gene files have been widely applied among different species and diseases. Such transcriptome research can not only testify to gene functions and structures comprehensively, but also disclose the specific pathophysiologic mechanism underlying disease. However, a majority of the existing research remains limited to the coding RNA. On its rising slope, the quantity and quality of research on ncRNAs remains insufficient. Circular RNAs (circRNAs), the novel type of endogenous ncRNAs, has widely attracted scientists' attention lately (7). Compared with linear RNAs, the structure of covalently closed loops determines the stability of circRNAs by providing resistance to the exonuclease (8). With the development of this technique, an increasing amount of research has revealed the features of a majority of circRNAs in mammalian cells. They are endogenous, conserved, stable, and abundant, making them ideal therapeutic targets or potential biomarkers for the future (9–12).

A previous study applying microRNA microarrays has detected several miRNAs with significant expressive changes upon hepatic IPC following IRI (13). Moreover, a recent study conducted by Ye Z et al. investigated differentially expressed cirRNAs in liver IRI (14). Using a microarray, Zhang P et al. went further, comparing cirRNAs in hepatic IRI with or without IPostC and regarded mmu_circRNA_005186 as having a potential protective role in IPostC (15). However, systematic detection of the dysregulated circRNAs and mRNAs in response to IPC intervention still remains to be elucidated. Here, we suppose that the expressive alteration of circRNAs may closely relate to the pathophysiologic mechanism of hepatic IRI and may contribute to IPC-mediated protection against hepatic IRI. Comprehensive application of microarray, quantitative real-time PCR (qRT-PCR), and a progressive analysis were applied to reveal the expressive changes of circRNAs in the model of hepatic IRI with or without IPC and to investigate the protective circRNAs related to hepatic IPC intervention. Our data may offer a new understanding of the mechanical basis of IPC and may provide a possible research target for prospective studies.

## Materials and Methods

### Experimental Animals

C57BL/6 male mice were obtained for our study. Each mouse weighed from 18 to 22 g and was fostered in a temperature-constant room and nourished by a standard chow pellet diet. Preoperative fasting was performed 12 h before the surgical procedure without water prohibition. The experiments were performed in accordance with the Institutional Animal Committee guidelines. All animal protocols were approved by the Institutional Ethics Committee.

### Model Construction and Experimental Groups

The hepatic IRI model was established by hepatic artery occlusion with clips as described previously (16). Briefly, after being anesthetized by pentobarbital (50 mg/kg body weight) and laparotomy, the mouse's portal vein and hepatic artery were isolated and temporarily clipped by atraumatic microvascular clip. After 1 h of ischemia to 70% of the liver, the vascular clip was wiped off to allow 6 h hepatic reperfusion. The ischemia extent was assessed by pale color of liver or pulselessness. Also, the recovery of the pale color and the pulse were considered to confirm valid hepatic reperfusion.

Three groups of the mouse model were constructed (*n* = 8 in each group): (1) sham group: sham model underwent portal vein and hepatic artery isolation with no occlusion; (2) IRI group: IRI model underwent IRI surgery without IPC; (3) IPC group: IPC was induced by 10 min of ischemia and 10 min of reperfusion before the intervention of IRI as reported previously (17). All the mice were sacrificed, and blood and organ samples were obtained at the end.

### Histological Analysis

To achieve hepatic injury, we collected hepatic samples from the different groups of mice and embedded them in paraffin. Then, 5 μm thickness consecutive sections were stained separately with hematoxylin and eosin (H&E), immunohistochemistry of F4/80, and Ly6G + and evaluated by microscopy.

### Analyses of Hepatic Injury and Cytokine Levels

Serum levels of alanine aminotransferase (ALT) and aspartate aminotransferase (AST) levels were tested using assay kits following the manufacturer's instructions (Nanjing Jiancheng). The levels of interleukin (IL)-6 and tumor necrosis factor (TNF)-α were measured using immunosorbent assay kits from the ENGTON Bio-engineering Limited Company (Shanghai, China) following the kit instructions.

### RNA Extraction

Tissue RNA was extracted from each hepatic sample using Trizol (Invitrogen, Carlsbad, CA, USA) following the instructions. The concentration and purity of RNA were accessed by a NanoDrop 2000 spectrophotometer (Thermo Scientific, MA, USA). Genomic DNA (gDNA) residual and RNA integrity were measured by denaturing agarose gel electrophoresis.

### Labeling and Hybridization

We selected three samples randomly from each group for microarray studies. RNA labeling and hybridization were done following the Agilent One-Color Microarray-Based Gene Expression Analysis construction. The microarray hybridization was applied following the manufacturer's instructions; five probes were used to increase the confidence of each transcript. To enrich circRNAs, total RNA of each sample was soaked with RNase R (Epicentre, Madison, WI, USA) to remove linear RNAs. Then, the Arraystar Labeling Kit (Arraystar, Rockville, USA) was used to transcribe circRNAs into fluorescent cRNA. Such cRNAs were then hybridized to the Arraystar circRNA array V_2_.0 (8x15K). The Agilent Scanner was applied to detect the arrays following washing the slides. In the mRNA study, the whole mRNA microarray (Agilent Technology, CA, USA) was conventionally applied.

### Microarray Information Analyses

Array data was processed by Agilent Feature Extraction software (version 11.0.1.1) and R software limma package. Low-intensity filtering was then applied after quantile normalization. The prominent different expressive circRNAs/mRNAs were sorted following the fold-change cutoff (fold change ≥ 1.5) or using volcano plot filtering. Distinctive circRNA/mRNA expression patterns were shown by hierarchical clustering.

### Pathway Analyses

Gene Ontology (GO) analyses (http://www.geneontology.org) were conducted to set up gene annotation built upon different organisms. Gene functions include cellular components (CC), biological processes (BP), and molecular functions (MF). The -log10 (*P*-value) represented GO score, denoting the abundance in different genes. Also, pathway clusters were harvested using Kyoto Encyclopedia of Genes and Genomes (KEGG) pathway analyses relying on existing molecular networks from various gene data. The -log10 (*P*-value) indicated the KEGG score, denoting the significant relationship with the putative pathway.

### Quantitative Real-Time Polymerase Chain Reaction (qRT-PCR)

The HiScript RT SuperMix for qPCR (+gDNA wiper) (Vazyme, Nanjing, China) was applied to reverse transcribe the total RNAs of each group following the protocol. QPCR using the SYBR Green kit (TaKaRa, Dalian, China) was applied to evaluate the expression levels of the circRNAs and mRNAs. Primers for mRNAs were routinely used. To amplify the circRNA junctions, we designed specific divergent primers based on the sequence obtained from the database “circBase” (http://www.circbase.org/). By using the 2-ΔΔCt method that normalizes against the expression of the β-actin gene, the circRNA and mRNA relative expressions were calculated. GenePharma (Shanghai, China) was applied to design, verify, and synthesize all primers. All sequences are as in Table 1.

**Table 1:** Primer sequences used for qRT-PCR analysis of circRNA and mRNA levels.

**Name**	**Primer F (5′−3′)**	**Primer R (5′−3′)**
mmu_circRNA_007095	CTATCTCTCAGAGGCAGGGG	TGGGTTTGAAGACAGCAACG
mmu_circRNA_017753	CGCAAGGTCACTGGAGGAAT	TCTTAACTGCCACACGATGC
mmu_circRNA_010415	AACTGGGCCGTGGCAATC	CACTGACACTCTTTCCCTCTGG
mmu_circRNA_000895	CTTGTCAGCCTCAGTGGGA	CTCAGAGGTCGTTTAGCTTGG
mmu_circRNA_001946	TCGGCGTTTTGACATTCAGG	GGAAGACCTTGGTACTGGCA
mmu_circRNA_027197	TGTTTGTGACCTCCCTCTCC	CAGAATCACGCCACACACTT
mmu_circRNA_010498	TGATTTCTTCTGTTATGGTGGCG	TGCATAGTCGTTGAAGAAGGC
Gclc	AGCCCTACGGAGGAACGA	CCTCTGGGTTGGGTCTGTG
Krt18	CTTGCCGCCGATGACTTT	TGCAGCCTTGTGATGTTGG
Hacl1	TCCCTCCAATGTGCCTCTT	CCTGCCTCAGCGAGTGTTG
Stim1	GCTGGCAAGAAGGCAATG	AAAGAAAGGAAGGGAGGTGAA
Phc3	TCGGGATGTGAGGATTAGGA	CGGGCAAAGAATGGATGAA
β-actin	AGAGGGAAATCGTGCGTGAC	CAATAGTGATGACCTGGCCGT

### Prediction of circRNA/miRNA Coactions

Arraystar's commercial miRNA coaction prediction software was applied to predict the circRNA/miRNA coaction. The database is based upon the existing online informatics tools miRanda (http://www.microrna.org/) and TargetScan (http://www.targetscan.org/). The Arraystar's miRNA scores of support vector regression (mirSVR) were used as a basis to rank miRNAs, and the miRNAs ranking the highest were considered for further analysis.

### circRNA–miRNA–mRNA Pathway Prediction

Arraystar's software was applied to search circRNA_017753 MERs and select top potential target miRNAs based on match sequences. Then, top target miRNAs for the circRNA were selected according to the databases miRDB (http://mirdb.org/) and TargetScan. Based on the ceRNA theory, the direction of circRNA and mRNA changes in the same orientation. To reveal the beneficial mechanisms of the circRNA_017753 in hepatic IPC, we identified predicted downstream mRNA targets in the IRI group and elevated in the IPC group and constructed a Venn diagram. In the end, we selected the overlaying mRNAs and constructed the circRNA–miRNA–mRNA pathway.

### Statistics

In the microarray data, a circRNA/mRNA fold change ≥ 1.5 was selected, and *P* < 0.05 was conventionally regarded as significant. GraphPad Prism 5.0 (GraphPad Software, CA, USA) was applied to analyze other data, and all data were indicated as the mean ± standard deviation. One-way analysis of variance followed by the Student–Newman–Keuls test were applied to compare data with normal distribution. The Kruskal–Wallis test and the Wilcoxon rank sum test with Bonferroni adjustments were used to compare data with nonnormal distributions. Means between two groups were compared by a two-tailed Student *t*-test. *P* < 0.05 was regarded as significant.

## Results

### Evaluation of Hepatic IRI and Hepatic Protection by IPC

**Figure 1:**
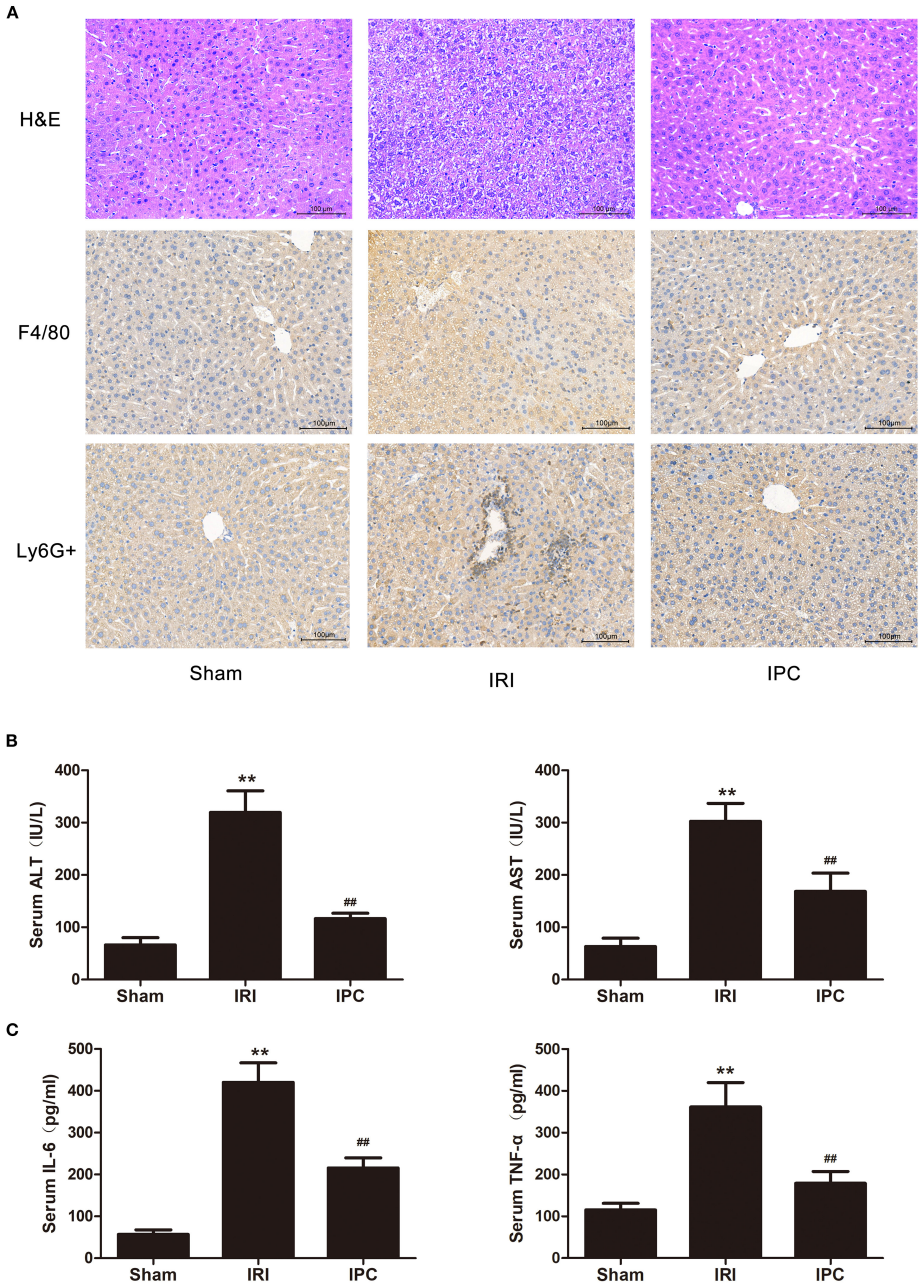
The IPC model is suitable for investigating mechanisms of protecting the liver against IRI. **(A)** H&E staining (up), immunohistochemistry of F4/80 (middle), and Ly6G + (down) images are shown for the hepatic histopathological alteration (magnification × 200). **(B)** Serum levels of ALT and AST. **(C)** Serum levels of IL-6 and TNF-α. Data are expressed as the means ± SD, *n* = 8. ^**^P <0.01 compared with the sham group; ^*##*^*P* <0.01 compared with the IRI group.

The blood index of hepatic injury after hepatic IRI with or without IPC were tested to identify the beneficial effect of IPC in the study. As presented in our H&E staining, liver IRI induced a marked hepatocellular necrosis. In contrast, mice with IPC presented with only minor signs of ischemic congestion and necrosis. Our histochemical evaluation of F4/80 showed an intense inflammatory process occurring in ischemic parenchyma and alleviated levels in IPC. Also, the immunohistochemistry of Ly6G + showed a decreased level of infiltrating Ly6G + cells (a neutrophil plasma membrane biomarker) comparing the IPC and IRI groups (Figure 1A). The hepatic injury by IRI and the beneficial effect of IPC were further identified by serum examination. Serum ALT and AST were upregulated after hepatic IRI, and IPC remarkably decreased serum transaminase levels (Figure 1B). In addition, IPC ameliorated the TNF-α and IL-6 increase caused by IRI (Figure 1C). The results indicate that the current IPC intervention is effective enough to protect the liver against IRI. Hence, the model is suitable for comparing the circRNAs among IRI with or without IPC intervention.

### Comparison of circRNA and mRNA in Hepatic IRI and IPC

**Figure 2:**
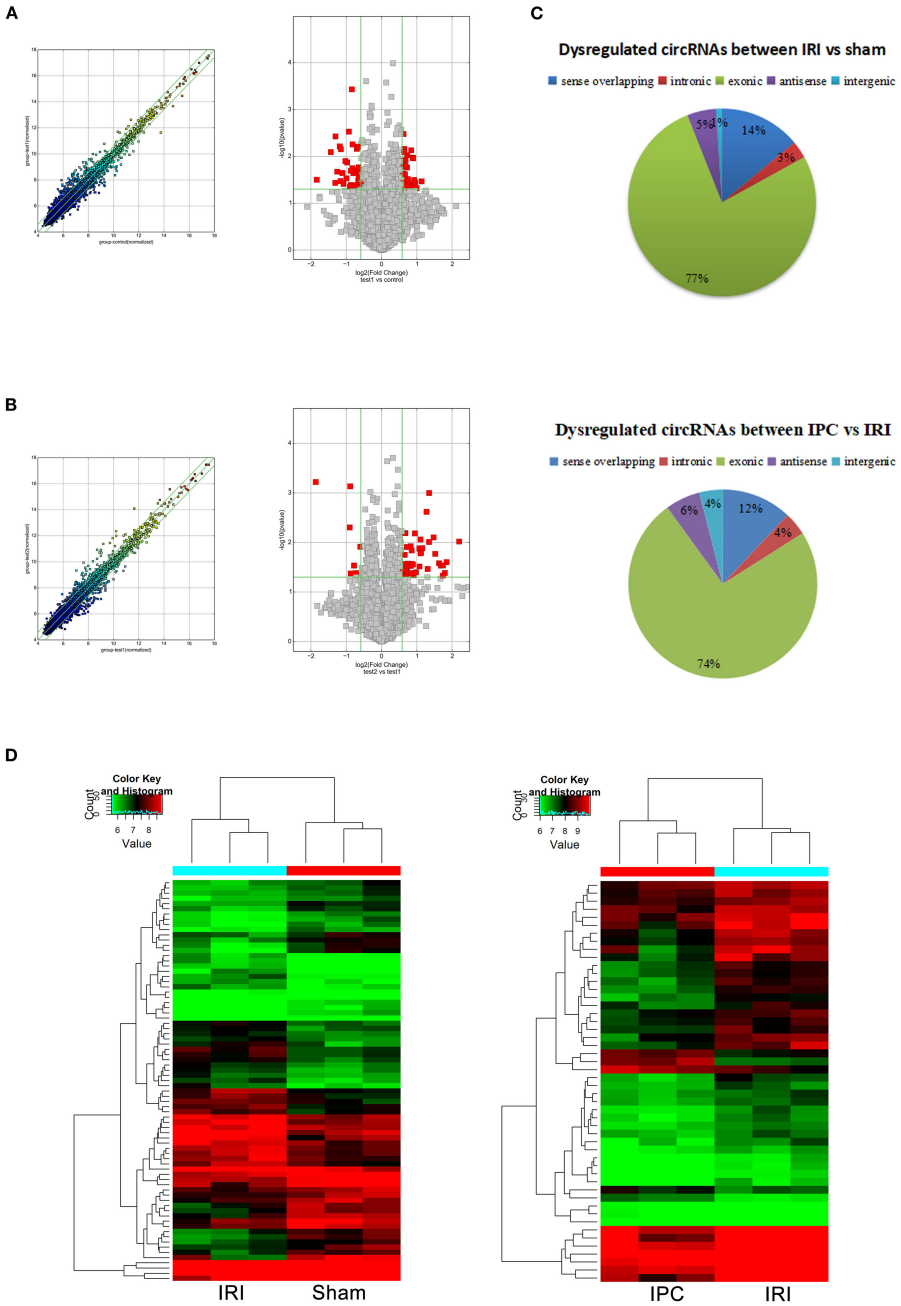
CircRNAs comparing the IRI and sham groups and between the IPC and IRI groups. The left (scatterplots) and right (volcano plots) show the alteration of circRNA expression between IRI and sham groups **(A)** and between IPC and IRI groups **(B)**. In the scatterplot, the values on the *X*- and *Y*-axes are the log2 scaled signals of samples. Fold change is represented by the green lines. The circRNAs outside the range formed by the upper and lower green lines are those with the fold change ≥ 1.5 between the compared groups. In the volcano plot, the vertical green lines represent a 1.5-fold change although the horizontal green line corresponds to a *P*-value of 0.05. The red points in the volcano plot represent the significantly altered circRNAs with *P*-value <0.05. **(C)** Different subgroups of significantly altered circRNAs according to their effects and position. **(D)** Heat maps of circRNA profiles from the microarray data. The color scales represent expression values. Red represents high expression, and green indicates low relative expression. Each row of colored boxes indicates a single circRNA, each column indicates a single sample.

**Figure 3:**
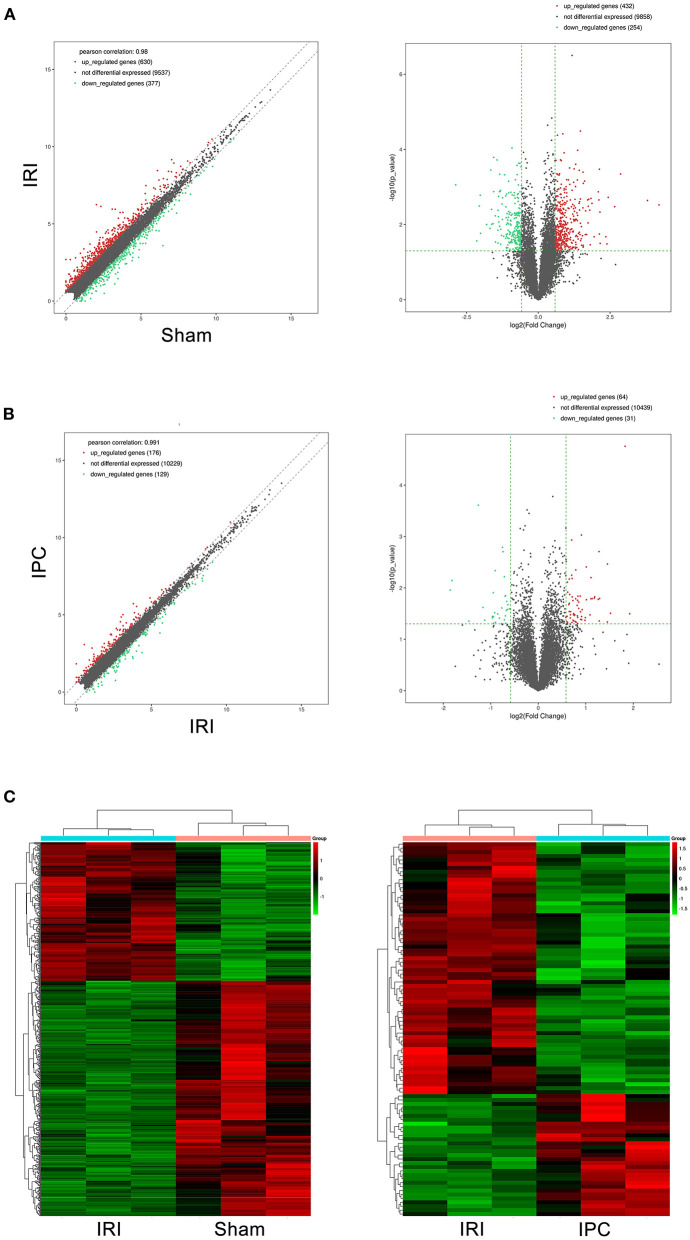
mRNAs comparing the IRI and sham groups and between the IPC and IRI groups. The left (scatterplots) and right (volcano plots) show the alteration of mRNA expression between IRI and sham groups **(A)** and between IPC and IRI groups **(B)**. In the plot, red and green points represent significant altered mRNAs (fold change ≥ 1.5, *P*-value < 0.05), respectively. **(C)** Heat maps of mRNA profiles from the microarray data. The color scales represent expression values. Red represents high expression, and green indicates low relative expression. Each row of colored boxes indicates single circRNA; each column indicates single sample.

To compare circRNA expression in the (1) sham, (2) IRI, and (3) IRI + IPC groups, we applied microarray analysis for circRNA and mRNA expression profiles of three samples in each. About 10,535 circRNA and 10,654 mRNA targets in the liver were spotted by microarray. Among them, 77 circRNAs and 686 mRNAs in the IRI group altered more than 1.5-fold compared with those in the sham group (*P* < 0.05). To be precise, 39 circRNAs and 432 mRNAs were increased, and 38 circRNAs and 254 mRNAs were decreased compared with the sham group (Figures 2A, 3A). Moreover, as a result of IPC intervention, a total of 50 circRNA and 95 mRNA alterations with significance (fold change ≥ 1.5, *P* < 0.05) were detected, in which 43 circRNAs and 64 mRNAs increased, 7 circRNAs and 31 mRNAs decreased in the IPC group when compared with IRI (Figures 2B, 3B). Based on fold change, we summarize the top 10 altered circRNAs in Table 2 and the top 10 altered mRNAs in Table 3. We then classified the significant altered circRNAs into various groups. Comparing the altered circRNAs in the IRI and sham groups, there were 77 exonic, 14 sense overlapping, 3 intronic, 5 antisense, and 1 intergenic and 74 exonic, 12 sense overlapping, 4 intronic, 6 antisense, and 4 intergenic when comparing the IPC and IRI groups (Figure 2C). Also, by using hierarchical clustering, we were able to cluster the differentially altered circRNAs and mRNAs of comparison groups and visualize the expression profiles. As indicated by the data, circRNAs and mRNAs in the IRI group were dramatically different from those of the sham group although IPC intervention significantly altered the IRI-induced expressions (Figures 2D, 3C).

Table 2Top 10 significantly dysregulated circRNAs and mRNAs ranked by fold change between I/R and sham groups.

**Table:** 

**CircRNA**					
**Name**	**circRNA_type**	**GeneSymbol**	**Fold Change**	**Regulation**	***P*****-value**
mmu_circRNA_29990	Exonic	Bach1	2.195	Up	0.034
mmu_circRNA_29992	Exonic	Bach1	1.987	Up	0.048
mmu_circRNA_32165	Exonic	Chka	1.933	Up	0.035
mmu_circRNA_22310	Exonic	Slc41a2	1.906	Up	0.043
mmu_circRNA_35001	Exonic	B4galt5	1.892	Up	0.032
mmu_circRNA_39714	Exonic	Wdr95	3.566	Down	0.031
mmu_circRNA_34157	Exonic	Qser1	2.707	Down	0.008
mmu_circRNA_000113	Antisense	Rian	2.482	Down	0.037
mmu_circRNA_29383	Exonic	Prodh	2.469	Down	0.004
mmu_circRNA_011844	Exonic	Inpp5a	2.390	Down	0.021

**Table:** 

**mRNA**			
**Gene Name**	**Fold Change**	**Regulation**	***P*****-value**
Gm3776	18.449	Up	0.003
Gsta1	13.939	Up	0.002
Serpina7	7.287	Up	4.50191E-04
Plscr1	6.328	Up	0.003
Tubb6	5.410	Up	0.002
G0s2	7.339	Down	0.001
Ppp1r3g	4.412	Down	0.027
Sftpa1	4.150	Down	0.003
Usp2	4.054	Down	0.002
Ppp1r3b	4.025	Down	0.017

Table 3Top 10 significantly dysregulated circRNAs and mRNAs ranked by fold change between IPC and I/R groups.

**Table:** 

**CircRNA**					
**Name**	**circRNA_type**	**GeneSymbol**	**Fold Change**	**Regulation**	***P*****-value**
mmu_circRNA_31583	Exonic	Sil1	4.585	Up	0.010
mmu_circRNA_000113	Antisense	Rian	3.586	Up	0.025
mmu_circRNA_37852	Exonic	Igsf21	3.465	Up	0.041
mmu_circRNA_19765	Exonic	Fam135a	3.335	Up	0.046
mmu_circRNA_38137	Exonic	Sema3c	3.334	Up	0.029
mmu_circRNA_38159	Exonic	Magi2	3.638	Down	0.001
mmu_circRNA_19091	Sense overlapping	Cdyl	1.866	Down	0.005
mmu_circRNA_41223	Exonic	Grik5	1.851	Down	0.001
mmu_circRNA_003780	Exonic	Cdyl	1.832	Down	0.042
mmu_circRNA_43573	Exonic	Banp	1.718	Down	0.029

**Table:** 

**mRNA**			
**Gene Name**	**Fold Change**	**Regulation**	***P*****-value**
Hist1h1c	3.800	Up	0.032
Kctd12	3.553	Up	1.75414E-05
Rab30	2.871	Up	0.031
Ankrd33b	2.743	Up	0.003
Ldlr	2.740	Up	0.046
Ppp1r3c	3.617	Down	0.011
Cyp2b10	3.529	Down	0.007
Nrg4	2.750	Down	0.044
Phc3	2.399	Down	2.44694 E-04
Ddit4	2.241	Down	0.024

### circRNA and mRNA qRT-PCR Verification

**Figure 4:**
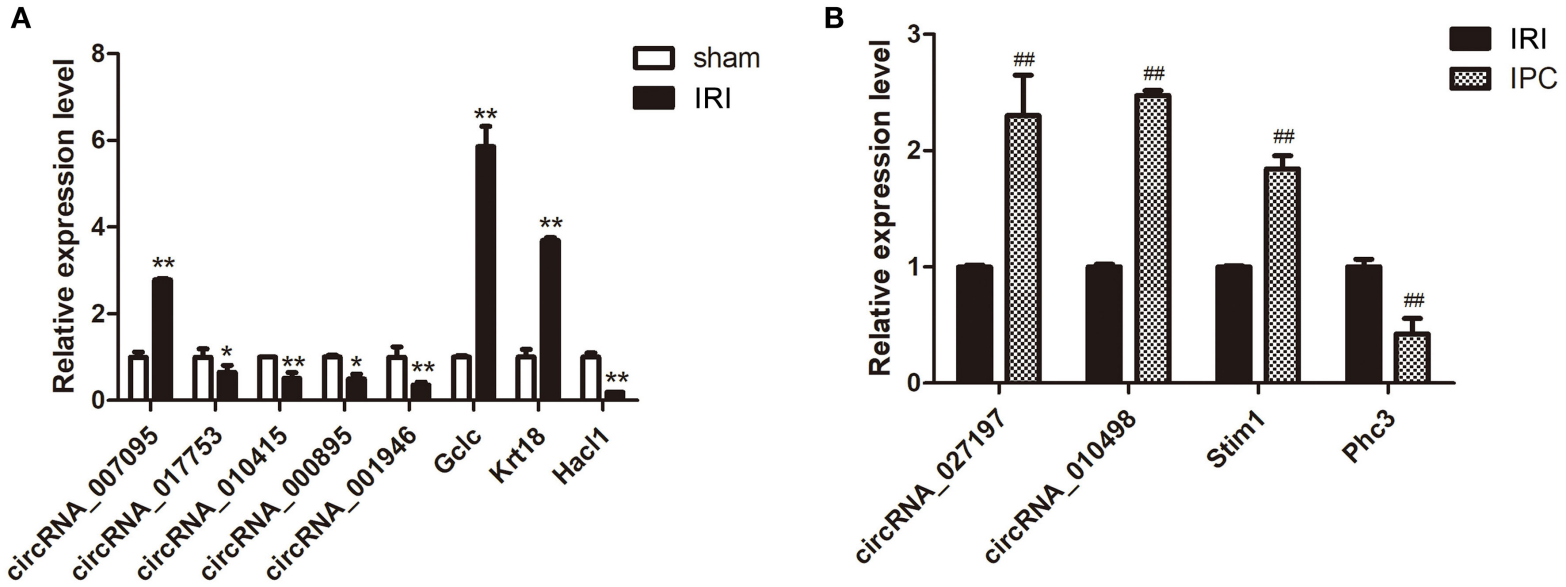
Selected circRNA and mRNA qRT-PCR verification between the IRI and sham groups **(A)** and between the IPC and IRI groups **(B)**. Data are expressed as the means ± SD, *n* = 6. ^*^P <0.05 compared with the sham, and ^**^P <0.01 compared with the sham group; ^*##*^*P* <0.01 compared with the IRI group.

qRT-PCR was further applied to confirm the microarray results. Seven circRNAs and five mRNAs were selected considering their fold change, *P*-value, and raw data. As qRT-PCR results show in Figure 4, the expression of circRNA_007095, Gclc, and Krt18 increased, and the circRNA_017753, circRNA_010415, circRNA_000895, circRNA_001946, and Hacl1 decreased enormously after hepatic IRI compared with the sham group. Otherwise, compared with IRI, IPC intervention remarkably elevated the expression of circRNA_027197, circRNA_010498, and Stim1, whereas Phc3 were reduced. The verified results are accordant with the data, indicating the dependability of our microarray profile.

### Analysis of Variously Expressed mRNAs

**Figure 5:**
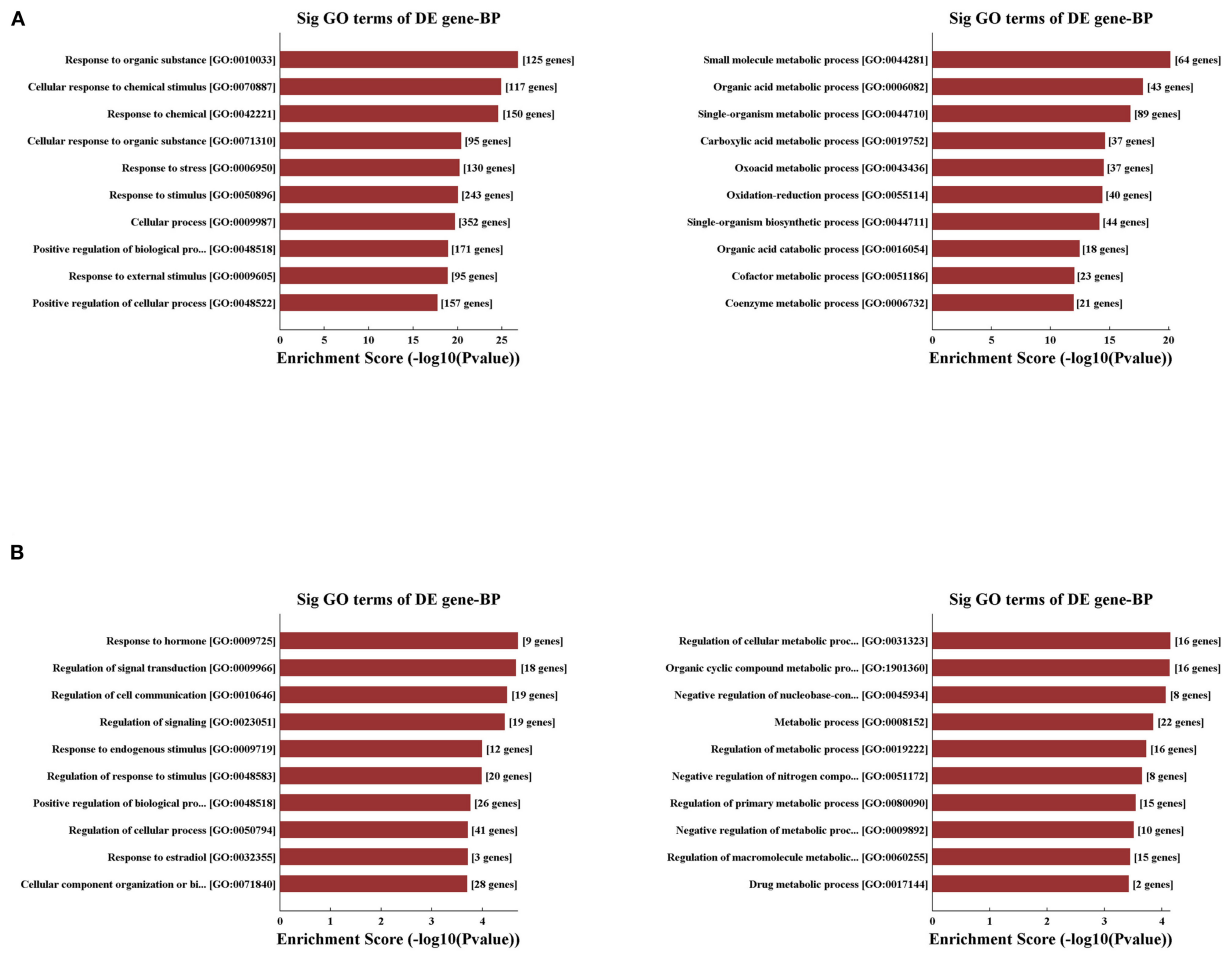
Variously expressed mRNA GO analysis. GO analysis between the IRI and sham groups **(A)** and between the IPC and IRI groups **(B)**. Top 10 up- (left) and downregulated (right) mRNAs were analyzed by GO analysis concerning biological processes.

Concerning that biologically similar genes share the same patterns of change, we decided to excavate the variations of coding gene expression among the IRI and IPC groups to reveal the behavior of these variously expressed circRNAs. GO enrichment analysis was used first for variously expressed mRNAs. In response to hepatic IRI, upregulated mRNAs were most related with response to organic substance and cellular response to chemical stimulus, and the downregulated mRNAs were most related to small molecule metabolic and organic acid metabolic processes (Figure 5A). Compared with the IRI, IPC mostly elevated the mRNAs related with response to hormone and regulation of signal transduction; meanwhile the downregulated mRNAs of IPC were most involved in regulation of cellular metabolic processes and organic cyclic compound metabolic processes (Figure 5B).

**Figure 6:**
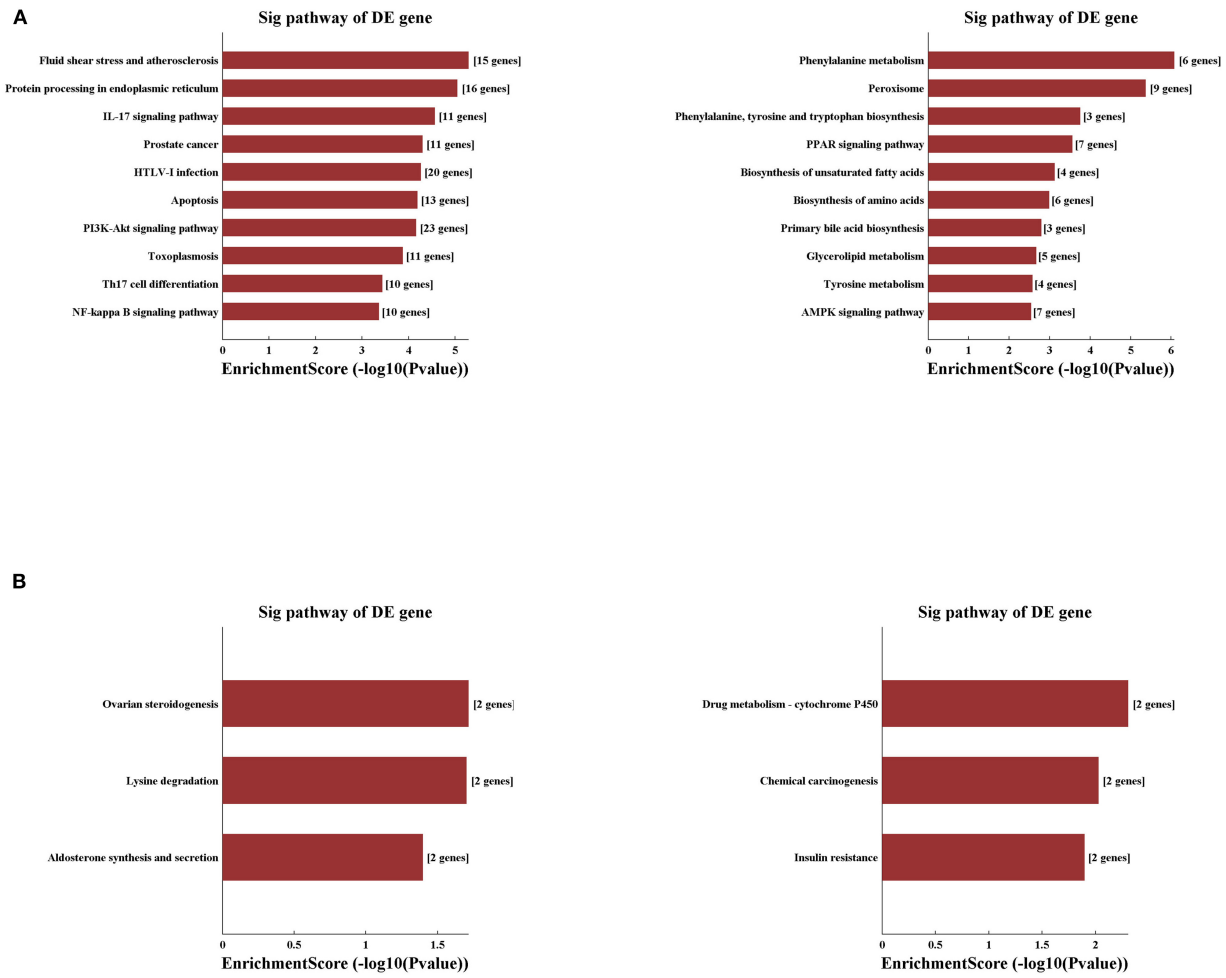
Variously expressed mRNAs KEGG pathway analysis. KEGG pathway analysis between the IRI and sham groups **(A)** and between the IPC and IRI groups **(B)**. Top 10 up- (left) and downregulated (right) mRNAs were analyzed by KEGG analysis.

Applying the pathway analysis of KEGG, we found that fluid shear stress and atherosclerosis and protein processing in the endoplasmic reticulum were involved in the upregulated mRNAs, whereas the pathways of phenylalanine metabolism and peroxisome were most involved in the decreased mRNAs in the IRI group (Figure 6A). Notably, pathways related to the inflammatory process and apoptosis were the most involved in the IRI process as listed, such as pathways of PI3K-AKT, NF-Kappa B, IL-17 signaling, and apoptosis. Moreover, compared with the IRI group, IPC can significantly upregulate mRNAs involved in the pathways of ovarian steroidogenesis and lysine degradation and downregulate the mRNAs related with drug metabolism-cytochrome p450 and chemical carcinogenesis pathways (Figure 6B). Our computational analysis results may contribute dramatically to the pathogenesis of hepatic IRI and IPC intervention.

### Identification of circRNAs Related to Hepatic Protection by IPC

**Figure 7:**
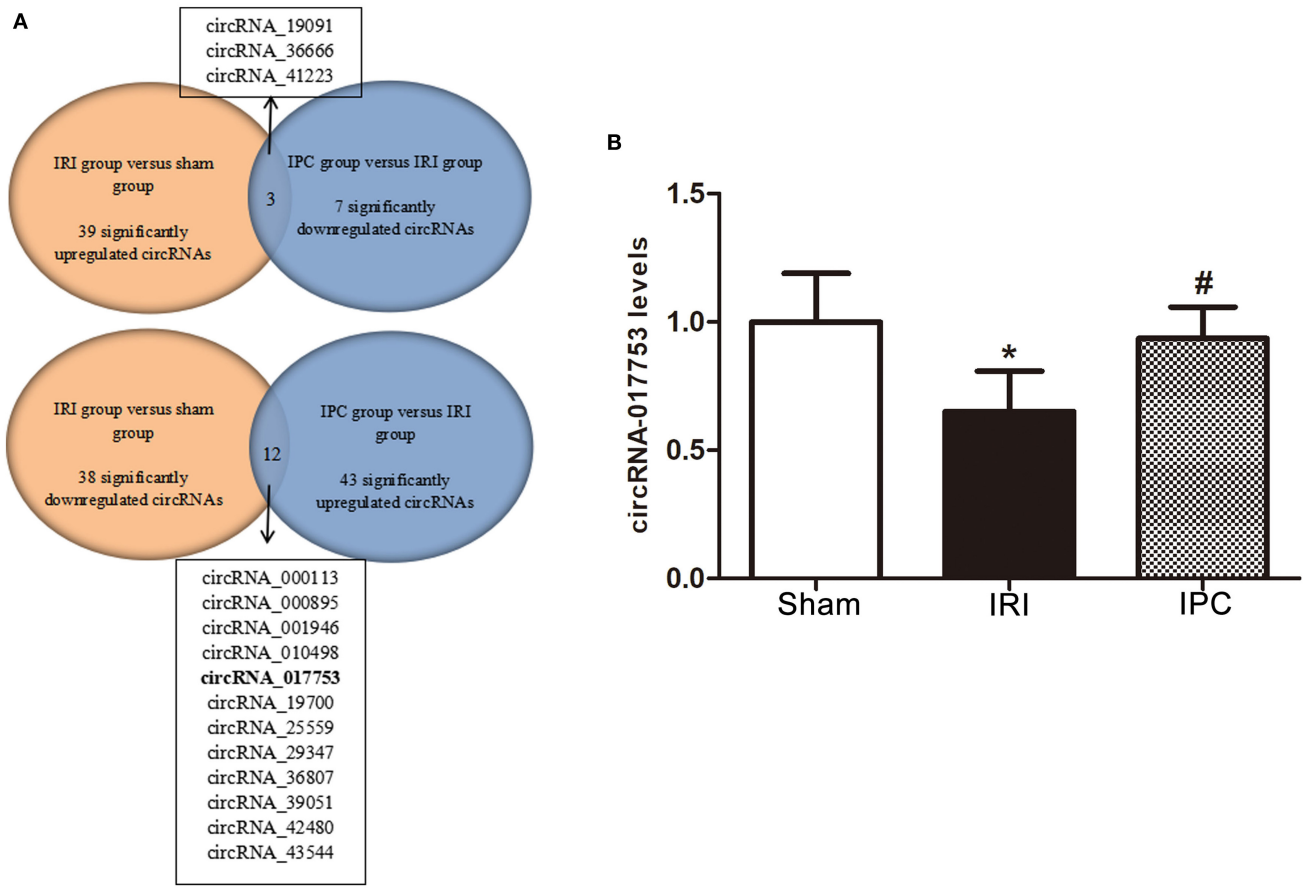
Identification of circRNAs related to hepatic protection by IPC and qRT-PCR validation. **(A)** The Venn diagram shows the circRNAs with an opposite direction of the alterations between the two comparison groups (sham vs. IRI and IRI vs. IPC). **(B)** qRT-PCR detected expression levels of circRNA_017753 in mouse liver among the three groups. Data are expressed as the means ± SD, *n* = 6. ^*^P <0.05 compared with the sham group; ^#^*P* <0.05 compared with the IRI group.

Concerning the protection effect of IPC intervention against IRI, we compared the circRNA alteration and direction of alterations of the three groups to reveal the potential relevance between circRNA changes and IPC protection. As a result, we sorted circRNAs that showed up with opposite alteration directions between the comparison groups (sham vs. IRI and IRI vs. IPC). Following this method, we selected three circRNAs that were upregulated in IRI but downregulated in the IPC group and 12 circRNAs that showed a completely opposite direction (Figure 7A). Concerning the type of circRNAs and data sources of circRNAs, we then selected only one circRNA, circRNA_017753, that was considered significatively related to the IPC protective effect. The gnomic locus of circRNA_017753 is on chromosome 17, and it is spliced from Mapk14. The circRNA_017753 expression level was then confirmed by qRT-PCR; IPC intervention can significantly restore the downregulation caused by hepatic IRI. Our data indicate that circRNA_017753 may play a protective role in hepatic IRI that arouses our desire to learn the potential role of circRNA_017753. In addition, we also compared our data with the microarray data of hepatic IPostC intervention in IRI (GSE117524) (15). Though circRNA_010498 does not show a comparative fold change to circRNA_017753 in our data, the intersection of research (fold change ≥ 1.5, *P*-value <0.05) indicates its potential significant protective effect in both IPC and IPostC intervention and is worth further study (Supplementary Figure 1).

### Prediction of miRNA and CircRNA–miRNA–mRNA Pathway for CircRNA_017753

**Figure 8:**
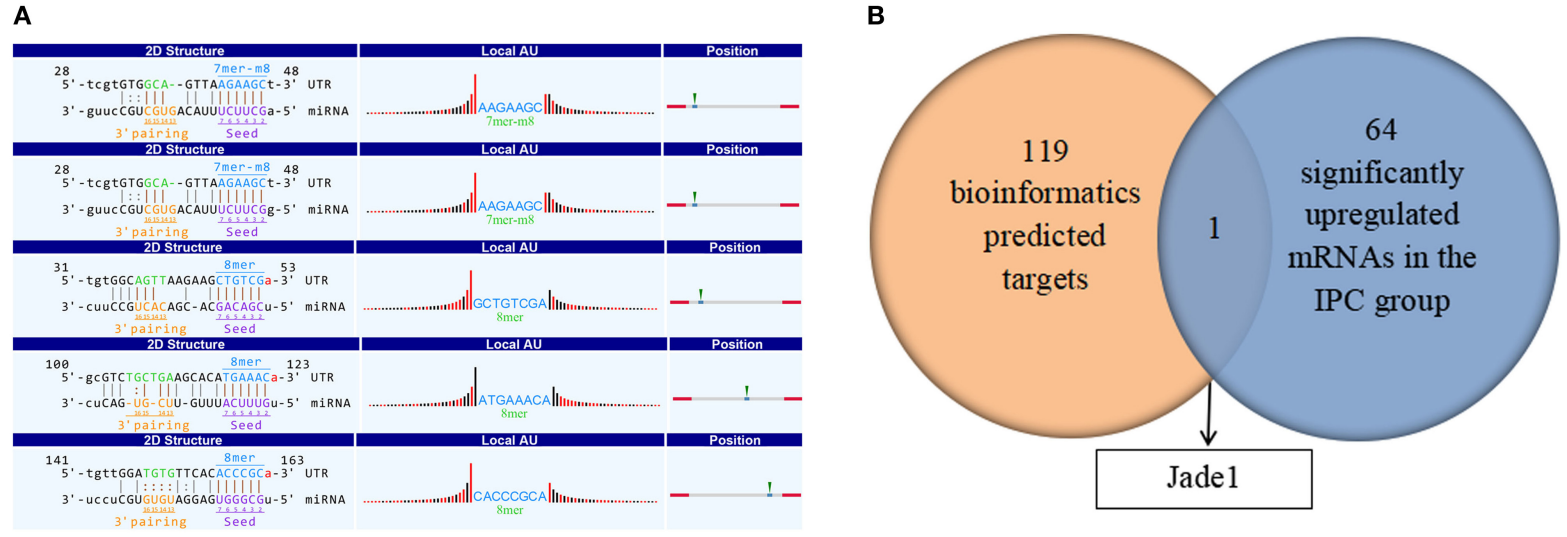
**(A)** Prediction of MiRNA and the circRNA–miRNA–mRNA pathway. Detailed structure of circRNA/miRNA interactions. The MRE sequence, miRNA seed type, precise base positions and target miRNA nucleotides are presented as annotation in 2-D structure column. Thirty nucleotides downstream and upstream the seed sequence are presented in “local AU.” Red bars present A/U, and black bars present G/C. The most likely relative MRE position are presented in position column. **(B)** Overlap of the predicted mRNA target related to circRNA_017753 and significantly upregulated mRNAs caused by IPC intervention.

It is well-known that circRNA regulates miRNAs by interacting with miRNA response elements (MREs). Such way of interaction can competitively suppress miRNAs' activity and is, thus, called miRNA sponges. To figure out the function of circRNA_017753, we applied Arraystar's prediction software, which combined TargetScan and miRanda databases. We predict and list the five highest ranking target miRNAs of circRNA_017753 and annotation of their circRNA/miRNA interactions (Table 4, Figure 8A).

**Table 4:** The identified circRNAs and its predicted miRNA response elements (MREs).

**CircRNAs**	**Alias (circBase)**	**Chrom**	**Gene Symbol**	**MRE1**	**MRE2**	**MRE3**	**MRE4**	**MRE5**
mmu_circRNA_017753	mmu_circ_0000737	Chr17 +	Mapk14	mmu-miR-103-2-5p	mmu-miR-103-1-5p	mmu-miR-196b-3p	mmu-miR-7675-3p	mmu-miR-6409

To further investigate the potential mechanisms of circRNA_017753 in IPC, we construct the possible circRNA_017753–miRNA–mRNA pathways. Applying Arraystar's miRNA prediction software, 119 genes were selected related to the 5 miRNAs mentioned (Supplementary Table 1). Because of the widely accepted ceRNA concept that circRNAs may positively regulate mRNAs, we paid more attention to the mRNAs downregulated in IRI but significantly inhibited by IPC. By establishing the intersections of 65 mRNAs significantly increased by IPC and 119 predicted mRNA targets connected to circRNA_017753, we identified only one overlapping mRNA, Jade1, and predicted three following circRNA–miRNA–mRNA signals: circRNA_017753–miR-218-5p–Jade1, circRNA_017753–miR-7002-3p–Jade1, and circRNA_017753–miR-7008-3p–Jade1. These regulatory ceRNA signaling pathways may play important roles in the mechanisms of IPC protection and deserve further study.

## Discussion

Hepatic IRI contributes significantly to organ damage in the surgical procedures of hepatic resections and liver transplantation. Such injury possesses a high mortality rate caused by an intense inflammatory process occurring in the ischemic liver. As presented in our research, not only did serum TNF-α and IL-6 increase in the IRI group, the evaluation of F4/80 and infiltrating of Ly6G + staining demonstrated an intense inflammatory process in IRI. IPC has been most investigated in the past decades as a life-saving intervention. Though widely applied clinically, the exact molecular mechanisms behind the protection effect of IPC still remain largely unclear. A majority of the existing research mainly focuses on protein-coding RNA while research on ncRNAs (long ncRNAs and circRNAs) remain insufficient. It was not until the mechanism of powerful miRNA sponges revealed circRNAs as a potential target for treatment and diagnosis scientists' attention was widely attracted. In our research, by utilizing circRNA microarray analysis, we first report circRNA alteration profiles induced by hepatic IRI and the protection intervention of IPC systematically. More importantly, through bioinformatic comparison of circRNA alteration and the direction of alteration, we identified one possible circRNA, circRNA_017753, related with hepatic protection by IPC intervention. By comparing the data with the previous microarray data of hepatic IPostC, we identified one possible circRNA, circRNA_010498, that may have a potential protective effect in both IPC and IPostC. Also, by integrated application of TargetScan and miRanda databases, we predicted three circRNA–miRNA–mRNA pathways that may take effect in the mechanisms of IPC protection against hepatic IRI.

In our research, the microarray profiles identified 77 circRNAs that were significantly altered (39 up- and 38 downregulated) after hepatic IRI, whereas a total of 50 circRNAs altered significantly due to the IPC intervention (43 up- and 7 downregulated). As previously reported, the majority of circRNAs arise from exons; our profiles identified that about 2/3 altered circRNAs were exonic (18). Though none of the circRNAs in our top dysregulated list have been reported before as the reason for nascent circRNA functional study, it is encouraging to see some mRNAs regarded as alternative transcripts of these circRNAs reported to take effect in different physiopathologic mechanisms related to the IRI process. For example, Bach1 is the alternative transcript of circRNA_29990 and circRNA_29992 (2 of the top 5 upregulated circRNAs in hepatic IRI), and its protein inhibits the transcription of HO-1 and related genes involved in the oxidative stress response by binding to Maf genome recognition elements (19). Bach1 deficiency may increase resistance to ischemic stresses by elevating HO-1 expression (20, 21). Several studies also reveal its important role in ischemic or oxidative damage (22–24). More importantly, Bach1 is identified to repress Wnt/β-Catenin signaling and angiogenesis in peripheral ischemic injury in a recent study (25). Magi2 is the transcript of circRNA_38159 (one of the top five downregulated circRNAs listed in IPC); it encodes scaffolding proteins binding to PTEN and is identified an important element in the ischemic injury of the central nervous system. As it is well-known that circRNAs may regulate its linear counterparts (26), the circRNAs listed may play crucial roles in hepatic IRI and IPC by regulating the transcription of the parent genes.

The data on dysregulated mRNAs are also inspiring. In the list of top 10 dysregulated mRNAs in hepatic IRI, G0s2 (G0/G1 switch gene 2) decreased the most among all downregulated mRNAs and is widely recognized as a direct activator of oxidative phosphorylation at the early phase of hypoxia (27, 28). A recent study just identified that its overexpression can alleviate ATP decrease in myocardial cells and increase their hypoxic resistance during ischemia (29), suggesting its crucial role in hepatic IRI. Also, we can identify some new clues on the protection mechanisms of IPC in our profile of dysregulated mRNAs between IPC and I/R groups. For instance, Ddit4 (DNA-damage inducible transcript 4), widely recognized as an autophagy regulator by negatively regulating mTORC1, is found downregulated in the IPC group (30). Our result is consistent with the previous research that Ddit4 is a novel protection molecule that prevents ischemic injury in hepatocytes (31). Also, the protective effect of Ddit4 is found to be vital in cardiac and cerebral IRI (32–34). All these consistencies prove the reliability of our profile and provide us the credibility of other listed mRNAs in the mechanisms of IPC protection against hepatic IRI, thus making it worth further study.

We can also profit from GO and KEGG pathway analysis of altered mRNAs, which may reveal the key processes of hepatic IRI pathogenesis. The data emphasize the crucial roles of the inflammatory process and apoptosis in the IRI process, such as pathways of PI3K-AKT, NF-Kappa B, IL-17 signaling, and apoptosis. Moreover, the pathways of the metabolic process and regulation of signaling are prominent when comparing the IPC and IRI groups, indicating that related genes may play vital roles in IPC protection mechanisms. Our observation was partially consistent with a previous study revealing the relationship with metabolic process and the IPC protective mechanism (35).

It is worth mentioning that, in our data, only one circRNA, circRNA_017753, was selected to have a relationship with the protective mechanisms of IPC with the most possibility. Our data on the microarray and qRT-PCR both confirm its decrease in the IRI group and upregulation with IPC intervention. We speculate that the decreased level of circRNA_017753 may reflect hepatic dysfunction during IRI, and IPC may alleviate the hepatic dysfunction by the circRNA adjustment. Thus, circRNA_017753 may play a crucial role in the protective mechanism of hepatic IRI and may be a possible therapeutic target of hepatic injury.

To make the analysis more comprehensive, we also compared our data with previous data on hepatic IRI and IPostC prevention (14, 15). Because of different experimental environments and model construction methods (such as reperfusion 4 h after ischemia 1 h by Zhang P et al.), though reported circRNAs, such as circRNA_005186, exhibit the same alteration trend in our study in both IRI and prevention, the fold change of these circRNAs does not meet our screening criteria (fold change ≥ 1.5, *P*-value <0.05). However, it is worth noting that, by constructing the intersection of our data and GSE117524, circRNA_010498 shows sufficient significance as a protective factor in both IPC and IPostC intervention.

It is widely recognized today that circRNAs regulate gene expression by serving as miRNA sponges. Based on sequence comparing and bioinformatic methods, we predicted the five potential candidate miRNAs for circRNA_017753 with the most possibility. Then, by applying prediction software that combines TargetScan and miRanda databases, we are able to construct three circRNA–miRNA–mRNA regulatory axes that may have a protective effect in IPC intervention. Even though three circRNA–miRNA–mRNA axes have not been previously reported, the predicted molecule of Jade1 was found to have a huge possibility to play a part in the hepatic IPC protection mechanism. As a component of the HBO1 complex, Jade1 was identified as a key regulator of apoptosis (36). In previous study, by promoting acetylation of histones, it functioned as a key regulator of cycle progression and redifferentiation in renal tubule regeneration after IRI (37). Further studies on Jade1 and related circRNA–miRNA–mRNA axes are in progress in our laboratory.

The current study has some limitations that should be admitted. First, our data was obtained in an animal model and may not fully represent human pathological pathways. However, there is a high degree of similarity between human and mouse research in circRNAs. As it is demonstrated in previous research that most circRNAs present to be conserved between mouse and human (38), most of the circRNAs detected in our microarray data are conserved and of great interest for human research. Second, our research focuses on integral hepatic parenchyma injury and IPC prevention, thus less consideration was put into the design of circRNA profiling in different hepatic zones. In the development of spatial transcriptomics and microarray methods, specific profiling for circRNA expression in different hepatic zones would be of great interest and worth further study.

## Conclusion

Our study for the first time delineates the expression data of dysregulated circRNAs and mRNAs in response to hepatic IRI and IPC intervention. Our profile and bioinformatic analyses provide numerous novel clues on the pathophysiologic mechanism of IPC protection. Several potential circRNA–miRNA–mRNA axes we predicted may offer promising targets for hepatic ischemic prevention and treatment.

## Data Availability Statement

The datasets presented in this study can be found in online repositories. The names of the repository/repositories and accession number(s) can be found below: GSE164367, https://www.ncbi.nlm.nih.gov/geo/query/acc.cgi?acc=GSE164367.

## Ethics Statement

The animal study was reviewed and approved by The First Affiliated Hospital, Zhejiang University School of Medicine.

## Author Contributions

XT, HX, LZ, and SZ designed the study. XT and YH performed most of the experiments. YL and ZY performed part of the experiments. XT, YH, and ZY analyzed the data. XT prepared and wrote the manuscript. All authors contributed to the article and approved the submitted version.

## Conflict of Interest

The authors declare that the research was conducted in the absence of any commercial or financial relationships that could be construed as a potential conflict of interest.

## Supplementary Material

Supplementary Figure 1 Identification of circRNAs related to hepatic protection by IPC and IPostC. The Venn diagram shows the possible protective circRNAs intersection of our data and GSE117524. (fold change≥1.5, *P*-value<0.05).
