# Supplementary figures and images for: Circular RNA Microarray Analyses in Hepatic Ischemia-Reperfusion Injury With Ischemic Preconditioning Prevention
Source: Front Med (Lausanne). 2021 Mar 8;8:626948. doi: 10.3389/fmed.2021.626948 (PMC7982475; doi:10.3389/fmed.2021.626948)

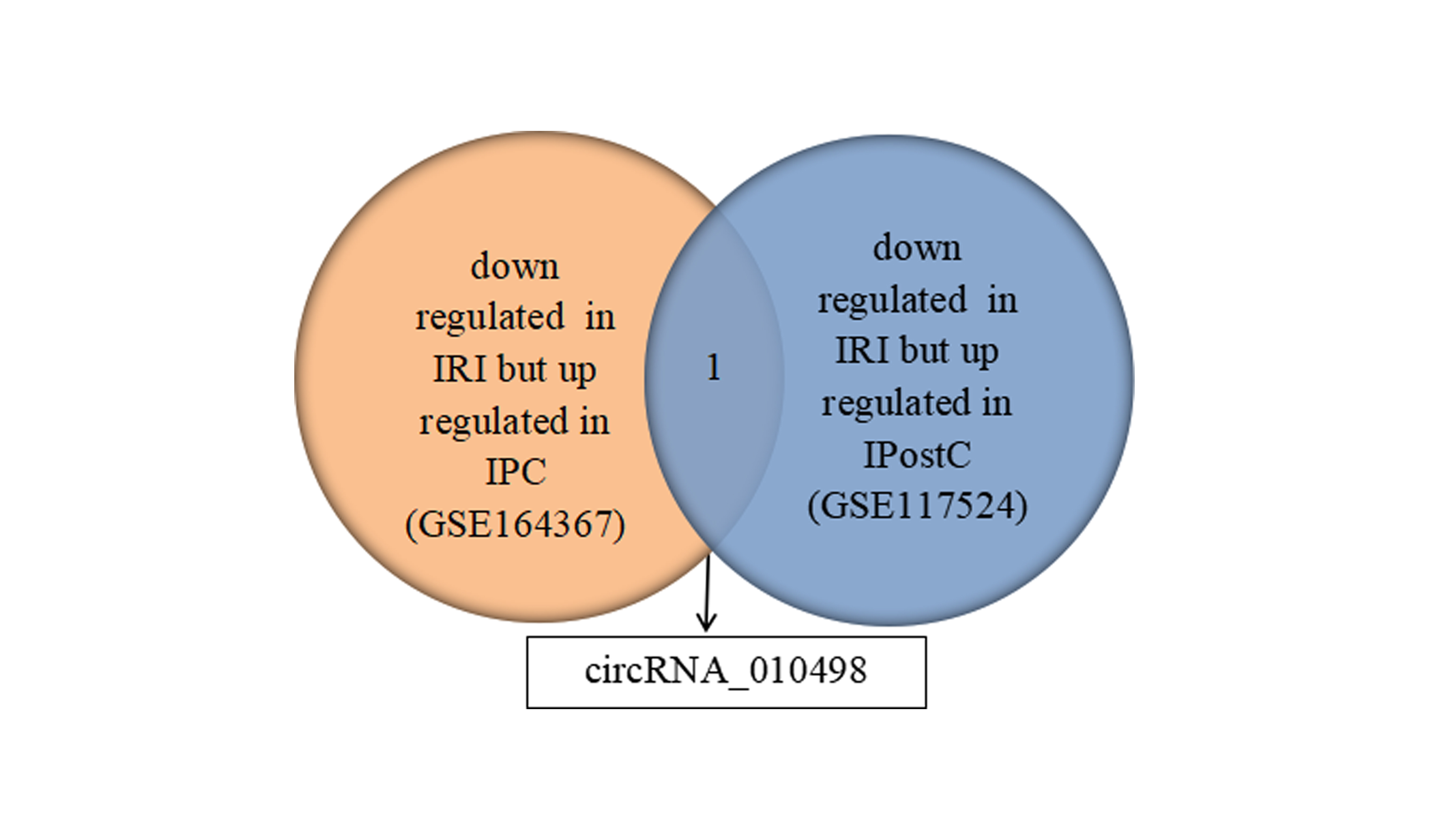

Supplement: Supplementary Figure 1 — Identification of circRNAs related to hepatic protection by IPC and IPostC. The Venn diagram shows the possible protective circRNAs intersection of our data and GSE117524. (fold change≥1.5, P-value<0.05). [file Image_1.jpg]
